# An Empirical Study on the Benefits Equity of the Medical Security Policy: the China Health and Nutrition Survey (CHNS)

**DOI:** 10.3390/ijerph17041203

**Published:** 2020-02-13

**Authors:** Huan Liu, Weidong Dai

**Affiliations:** School of Public Administration, Zhejiang University of Finance & Economics, Hangzhou 310018, China; zcliuhuan@126.com

**Keywords:** equalization of healthcare service, benefits equity, basic medical insurance, private medical insurance, medical assistance, preventive health care, Heckman two-stage selection model

## Abstract

*Background:* One of the fundamental objectives of the basic medical security system is to provide institutional guarantees for the appropriate medical needs of different groups. Among them, achieving fairness of benefits is the first principle of the system. This study aims to explore the benefit equity of preventive health care for different groups and the specific path to promote fairness. *Methods:* Based on the 2015 CHNS survey data, through the theory construction of benefit fairness in the basic medical insurance and using the two-stage IV-Heckman model, the paper analyzes the benefit fairness of the basic medical insurance in urban and rural China. *Results:* This study indicates that (1) the results of empirical and theoretical models are not consistent with the sample of the insured population. (2) As private medical insurance and medical assistance are restricted in the model, the reimbursement ratio of medical insurance in other income groups is all higher than the highest one. However, the coefficient is getting larger, with the lowest income group having the largest coefficient. After controlling for variables of disease and severity, the results suggest that the main impact path is hospitalization costs. (3) Taking the highest income group as a reference, the compensation proportion of preventive health care in other groups is higher, respectively, than the reference group, while the groups below middle income have a significant relationship with compensation for preventive health care. *Conclusions:* Supplementary private medical insurance and medical assistance have important protection functions for low- and middle-income populations. However, owing to the actual income threshold, the two groups cannot benefit from the medical security system. This result is still valid in the field of preventive health care. The increase of preventive health care expenditure reduces the cost of individual hospitalization, but the high-income group has emerged with more preventive health care expenditures, creating new unfairness.

## 1. Background

Based on the worldwide development process of the basic medical insurance system, the original intention of the system is to effectively fulfill the fundamental medical needs of every citizen. In the process of basic medical insurance reform, adhering to basic fairness is always the ultimate criterion for all counter attempts and a critical indicator to evaluate the success or failure of medical reform [1]. This study designs an equalization system for the payment and compensation mechanism of basic medical insurance and focuses on the fairness of the system guarantee to different income groups under equalization. Judging the current implementation of the basic medical insurance system in various countries, the main factors affecting the development are the too low compensation level of basic medical security and a non-equally distributed level of compensation in practice. Many documents have confirmed that medical insurance has played a positive role in family economy and anti-poverty through compensation for disease treatment costs [2,3]. Results from empirical research show that the absolute value elasticity of demand for medical services is generally less than 1, that is, in practice, the economic compensation mechanism of medical security is conducive to reducing the medical treatment of sick families [4,5]. It reduces the out-of-pocket medical expenses and burden of medical expenses. However, some scholars have found that medical insurance has no effect or even negative effects on that [6]. This study attempts to construct a theoretical framework on the equalization of basic medical security, which examines the fairness of the benefits in different income groups and explores the impact of supplementary private medical insurance and social medical assistance on the fairness of basic medical benefits in different income groups.

The system parity of basic medical insurance mainly refers to system design in the overall area, that all insured can pay the same premium, and that compensation has the same proportion, that is, the benefit of each insured person is equal to achieve equal rights [7,8,9]. However, the basic medical insurance system, which is designed in accordance with the equal design, does not emphasize absolute fairness of the result but is designed to promote equal access to health services for different income groups, such as accessibility to hospitalization. To ensure that more population with a low income have access to basic health services is the fundamental purpose of system construction. Therefore, from the perspective of equity, a necessary examination is whether the current system design has promoted a greater number of low-income groups to obtain better benefits from the system and improve their financial viability [10]. Norman Daniels believes that maintaining and achieving fair equality of opportunity is the basic principle of preventive health care, and he proposed a “10 fairness indicators” measurement system. Among them, Universal coverage and Participation and Minimizing nonfinancial barriers are the primary goals. In his view, the difference in health or disease will profoundly affect a person’s access to social resources, so a fair society should provide everyone with fair and equal opportunities. In order to achieve fairness, it is necessary to adopt a compulsory universal insurance system in health care to compensate for various inequalities caused by education, income, and socioeconomic status, etc. [11,12]. Finkelstein et al. [13] evaluated Oregon’s Medicaid and considered that Medicaid significantly increased the number of low-income participants utilization of preventive care and primary diagnosis and treatment, and self-borne medical expenditures have been significantly reduced. Jowett et al. [14] found that the Vietnamese medical insurance system has reduced residents’ out-of-pocket medical expenses by an average of 20%, and the benefits of low-income groups are significantly greater than those of the rich. However, some studies have found that medical insurance has not effectively reduced the residents’ medical burden. For example, Wagstaff et al. [15] and Lei et al. [16] and other studies on China’s new rural cooperative medical system found that the effect is not obvious. Previous studies have shown that low-income groups will benefit better than high-income groups, but the research conclusions are inconsistent. From the perspective of driving medical fairness indicators, applicability for medical insurance and its reimbursement ratio, and health care compensation have become the focal points of existing research.

Improving the assessment of the fairness of basic medical insurance would help provide reliable support for system optimization. However, in the evaluation of the institutional effect, it remains necessary to define fairness clearly and draw on the literature and main influencing factors that cause inequitable benefits of the group’s basic medical insurance, including, for example, objective factors, such as individual health and age and the differences in social factors, such as risk, income, and education [17,18,19,20]; among them, the social factors are the main cause of social inequality. Therefore, the definition of fairness in this paper emphasizes the differences between income groups in basic medical insurance, that is, different income groups are not provided with the same health rights despite being provided the same health insurance. The health right includes, for example, the incidence and total expenditure of hospitalization in different income groups, the total expenditure on preventive health care, the accessibility of hospitalization, and the fairness of medical insurance compensation mainly including the applicability of medical insurance and reimbursement ratio of medical insurance. Based on the theoretical framework of the benefit equity in basic medical insurance, and construction of a two-stage Heckman model [21], we analyze the benefit differences between different income groups under the concept of the equalized basic medical insurance system.

A reasonable basic medical insurance system must consider coverage and compensation level; among them, medical coverage should achieve full coverage, that is, equal accessibility for all income groups, and a compensation level that balances the welfare improvement and efficiency loss effects caused by medical insurance reimbursement [22,23]. The welfare improvement of basic medical insurance in this study refers to the smooth consumption of the insured, and the protection of the insured that does not lead to the reduction or replacement of other consumption levels resulted from illness. Loss of efficiency refers to the moral hazard of medical insurance and does not result in excessive health service demand because of the increase in the level of protection [24]. Therefore, in theory, equitable basic medical insurance should effectively compensate for the medical deficiencies of a low-income class and fulfill the welfare growth of the majority group. This balance should achieve the equity of medical resources and the accessibility of health services among different income groups.

Based on the current research on the fairness of basic medical insurance, we propose the following summary:

First, based on the actual compensation level of basic medical insurance (actual reimbursement ratio), the basic medical insurance affects the medical needs of the insured. To examine the fairness of medical insurance compensation, we should assess the impact of basic medical insurance coverage on individuals. For example, from the end of the basic medical insurance process, the actual medical insurance reimbursement level changes from the level of compensation to the individual health service use and health status changes [15,25,26]. Bai et al. observe that medical insurance increased the level of non-medical expenditure and the impact on lower-income families [27].

Second, the medical costs after the basic medical insurance is insured [28,29]. Most scholars have observed that participation in basic medical insurance does not significantly reduce the actual medical expenditure of the insured [30].

Third, the fairness of basic medical insurance compensation from the perspective of individual health and income. Achieving fairness in basic medical services is a basic task in the reform of the medical and health systems in countries worldwide and has received increasing attention from the academic world. Scholars have studied the differences in the fairness of the basic medical insurance systems in typical countries and regions, such as the perspective of the relationship between national income and health inequality in different countries (or regions) [20]; the benefit of the difference between income groups was studied from the perspective of basic health service use and medical insurance compensation level [31,32].

Fourth, the fairness of basic medical insurance from the perspective of institutional optimization and improvement, such as the effect before and after the implementation of regional basic medical insurance [33]. To promote the fairness of the health care system, some scholars explore its compensation effect on vulnerable groups [34].

Although the current research on the fairness of basic medical insurance has achieved some progress, many shortcomings remain. The main performances are as follows:

First, in the studies on the actual compensation level of basic medical insurance, more attention is paid to the comparison between the effect before and after the implementation of the policy compared with different incomes within the insured group.

Second, the compensation fairness of basic medical insurance is discussed based on medical costs. Most researchers do not effectively distinguish the group differences in medical burden changes, but notably, the accessibility of health services to vulnerable groups affects their level of compensation [10].

Third, quantitative research is rare. Although scholars have the perspective of quantitative research, defects are observed in the basic theoretical framework, and the assumptions are too idealistic and lack practical significance. In terms of research indicators on the fairness of basic medical insurance, scholars often use the concentration index and Gini coefficient to examine the compensation fairness of medical insurance. The non-parametric method has advantages for unfair measurement, but it ignores the microscopic theoretical basis and lacks good policy guidance significance.

## 2. Methods

### 2.1. Theoretical Framework and Research Hypothesis

The article focuses on the unfairness of health care benefits caused by different income levels under the same conditions in medical needs. As a health-induced demand, the needs for health services directly respond to individual or family’s medical needs. Therefore, drawing on the model setting of Cutler and Zeckhauser [35], we implement the corresponding theoretical model according to the “equalization” of the medical insurance system and then explore whether the current medical insurance system has achieved equal benefits for participants with different incomes.

We assume that the utility obtained by the individual medical service is U(x,c); thus, the problem of maximizing the patient’s medical expenditure can be expressed as follows:

(1) $$\underset{c}{\max}\;HU^{h}\left(y,0\right)+\left(1-H\right)U^{s}\left(y-c,c\right)\;s.t.\;c\leq y$$

Among them, *x* is the consumption of the individual, which we define as y−c. c is medical expenditure or preventive health service consumption, and y is the total income of the individual. The total income here includes the current total value of individual wealth. Therefore, the constraint is that the medical expenditure is less than or equal to the total income of the individual. H indicates the probability of maintaining health, and (1−H) indicates the probability of disease occurrence. $U^{h}$ represents the utility of the individual health. We assume that the marginal utility of the individual medical expenditure is always zero; thus, the corresponding individual medical expenditure in the healthy state is also zero. $U^{s}$ indicates the utility of the individual when he or she is sick. The marginal utility of the medical expenditure when the individual is sick is greater than zero. Therefore, the individual optimizes his or her medical expenses and maximizes the marginal utility of medical expenditure.

The marginal utility of an individual optimized health service consumption (medical expenditure) should be equal to the marginal utility of other consumer expenditures that achieve an overall consumption balance. If the optimal health service consumption is $c^{*}$, the corresponding equation on health equality is

(2) $$U_{1}^{s^{\prime }}(y-c)=U_{2}^{s^{\prime }}(c)$$

In Equation (2), $U_{1}^{s^{\prime }}(x)$ represents the marginal utility of other consumption, $U_{2}^{s^{\prime }}(c)$ represents the marginal utility of medical consumption. Equation (2) is the derivation of $U^{s}(x,c)$, which is maximizing the total utility of the individual. It is a condition for maximizing the utility of medical consumption when sick. Here is an equilibrium condition that considers the utility of individual consumption. $U_{1}$ and $U_{2}$ are utility numbers for medical and non-medical consumption in the context of illness. According to the stability characteristics of the marginal utility of money, the aforementioned assumptions are not completely in line with reality. Therefore, the quasi-linearization adjustment of Equation (2) can be performed, namely,

(3) U(y−c,c)=y−c+H(c)

H′(c) is the derivative of H(c) in Equation (3), that is, the first-order derivative of the health function under a given medical consumption. Among them, the utility of medical expenditure to individuals conforms to the concave function, and the conditions are met: $H^{\prime }(c)>0$, $H^{\prime \prime }(c)<0$, $\lim_{c\to 0}H^{\prime }(c)\to +\infty$, and $\lim_{c\to +\infty }H^{\prime }(c)\to 0$.

We assume that the individual expenditure from non-medical consumption is the same as the marginal utility of the money, and all are unchanged, that is, the optimal marginal utility of consumer spending is 1. Therefore, for individuals who do not participate in medical insurance, when suffering from disease, their optimal medical expenditures fulfill the first-order conditions: $H^{\prime }(c^{*})=1$; among them, when individual income is low, all the income cannot fulfill the optimal medical expenditure. That is, when $y<c^{*}$, $H^{\prime }(c)>1$, that is, the income elasticity of medical expenditure is greater than 1, this refers to the optimal medical consumption expenditure, which is different from the constraint conditions under the maximum medical expenditure of Equation 1. The marginal cost of the health service is decreasing. According to the research, the income elasticity of medical expenditures is different, mainly in the following: under the large coverage of medical insurance, the income elasticity of medical expenditure is close to zero or negative [36,37,38]; at low levels of protection, the income elasticity of medical expenditures increases to greater than 1, that is, the increase in medical expenditure is higher than the increase in income [39,40]. Therefore, the adjustment of Formula (3), highlighting the budget constraint, is the dominant path of income to medical expenditure.

The aforementioned was used to consider the relationship between unincorporated individual income and medical expenditure and then further consider the relationship between the two under the coverage of medical insurance. An assumption is that under the coverage of universal medical insurance, the health service market can be divided into two groups: a $n_{l}$ low-income group with income $y_{l}$ and high-income group $n_{h}$ with income $y_{h}$. And they are consistent with this formula: $y_{l}<rc^{\prime }+i<y_{h}$. Under the government-sponsored medical insurance system, the insurance premium rate is i, assuming a co-payment proportional payment system, a reimbursement ratio of r, the medical insurance that pays only (1−r) times the total medical expenditure. Based on the aforementioned assumptions, the problem of maximizing medical expenses under the coverage of medical insurance is

(4) $$\underset{c}{\max}\;y-i-rc+H(c)\;c.t.\;rc+i\leq y$$

According to equation (4), taking into account the insurance conditions, the medical expenditure under the health status is derived. Among them, since the income and rate are externally determined, the derivative is 0, and the medical expenditure and reimbursement ratio is under the insurance. It will affect health status and medical expenditures, so it can be obtained $c^{\prime }={H^{\prime }}^{-1}(r)$ after differentiation, that is, the optimal medical expenditure conditions are that marginal medical consumption is the inverse of the marginal health and reimbursement ratio. The first-order Solution (4) can be covered by medical insurance, and the optimal medical expenditure for the high-income group is $c^{\prime }={H^{\prime }}^{-1}(r)$. Because r<1, there must be $c^{\prime }>c^{*}$, where $c^{\prime }-c^{*}$ can measure the moral hazard caused by the co-payment insurance system. For people with a low income, budget constraint tightening means that individuals do not have an income insufficient to pay for medical expenses. Therefore, the marginal utility of medical expenditure is greater than the marginal cost of money, and the medical expenditure of people with a low income is the total income after payment in which the premium is $y_{l}-i$. Under the “equalization” medical insurance system, the insurance premium rate of each individual can be expressed as follows:

(5) $$(1-p)(1-r)[n_{h}c^{\prime }+n_{l}\frac{y_{l}-i}{r}]=(n_{l}+n_{h})i$$

The left side of Formula (5) represents the total medical expenditure, and the right side represents the total income of medical insurance; among them, the amount of medical insurance compensation for each high-income earner is $(1-r)c^{\prime }$, and the compensation amount for the low-income is $\left[(1-r)/r](y_{l}-i\right)$. Considering that in the process of “equalization” of the medical insurance system, there is an equivalent premium subsidy for every insured person. To simplify the analysis, the article does not consider government subsidies. After adjusting Formula (5), we obtain

(6) $$i=\frac{\left(1-r)(1-p)(c^{\prime }n_{h}+n_{l}\frac{y_{l}}{r}\right)}{n_{l}+n_{h}+(1-r)(1-p)\frac{n_{l}}{r}}$$

Under the “equalization” medical insurance system, an individual pays the same premium. Therefore, according to the relationship between the medical expenses and high-income people and people with a low income covered by medical insurance, two hypotheses are proposed:

**Hypothesis** **1 (H1).** *The personal medical expenditure of low-income participants increases linearly with the increase of income constraints, and the income elasticity of medical expenses of high-income participants is small.*

Combined with the aforementioned analysis, when there is no medical insurance, the individual medical expenditure changes (depreciation) to the former $c^{*}$. When the income is less than $c^{*}$, the medical expenditure increases with the increase in income; if the income is greater than $c^{*}$, at the time, medical expenses will not increase. With coverage of medical insurance, the new medical expenditure breakpoint changes; generally, it will reduce from $c^{*}$ to $rc^{\prime }+i$, and the decline is determined by the protection level r of medical insurance. The medical expenses paid by the high-income group are $rc^{\prime }$, and the medical expenses paid by low-income are $y_{l}-i$, of which $rc^{\prime }>y_{l}-i$. From the perspective of the co-payment system of medical insurance, the more medical expenditures, the greater the medical compensation. The medical compensation received by high-income earners is $(1-r)c^{\prime }$, and the low-income have only the $(y_{l}-i)(1-r)/r$ of the compensation ratio. Considering the relationship between fair insurance rates and the benefits between the two groups, the fair insurance price for the high-income is $(1-p)(1-r)c^{\prime }$, which can be obtained by gradual reduction:

(7) $$(1-p)(1-r)c^{\prime }=\frac{(1-p)(1-r)(n_{l}+n_{h})c^{\prime }}{n_{l}+n_{h}+(1-p)(1-r)\frac{n_{l}}{r}}>\frac{\left(1-p)(1-r)(\frac{y_{l}}{r}+c^{\prime }n_{h}\right)}{n_{l}+n_{h}+(1-p)(1-r)\frac{n_{l}}{r}}=i$$

The fair insurance premium for the low-income is a. When people with a low income or high income belong to the same fair insurance framework, Formula (7) is also established as follows:

(8) $$(1-p)(1-r)y_{l}<\frac{\left(1-p)(1-r)(\frac{y_{l}}{r}+c^{\prime }n_{h}\right)}{n_{l}+n_{h}+(1-p)(1-r)\frac{n_{l}}{r}}=i$$

Based on the aforementioned analysis, the other hypothesis arises:

**Hypothesis** **2 (H2).** *Under the “equalization” medical insurance system, the benefits of high-income insured are better than those under the fair medical insurance system, and low-income insured are inferior*.

Hypothesis 2 implies that “equalization” medical insurance is a “positive benefit” for high-income participants, and the medical insurance compensation exceeds the insurance premium; for low-income participants, there is “negative benefit”, and the payment as the cost of medical insurance exceeds the expectation of medical insurance compensation. That is, under the system, there are people with a low income who “reverse” subsidies to high-income earners.

### 2.2. Models

Based on the differences in the benefits of medical insurance for individuals with different incomes, we observe more cases in which the insured individuals have a reimbursement amount of 0 under medical insurance when they suffer from disease. A general belief is that this type of phenomenon is caused by factors other than the medical insurance system (e.g., the deductible line), that is, whether the compensation from medical insurance occurs or not, the compensation is independent of each other. The existence of these two reasons results in the non-normal distribution of the random error of the sample, and the direct ordinary least squares regression has an estimation bias. Therefore, two estimation models are used to be corrected in order to avoid the estimation bias. First, we build the first phase of the selection model:

(9) Probit(Reimbursement_dummyn=1|Income,X)=a0+∑m=15βm×Incomenm+Xnη+εn

In Equation (9), Reimburse_dummyn represents the binary variable of the medical reimbursement amount. Here, we define the medical reimbursement amount to be greater than 0, and 1 indicates that medical reimbursement is generated; the medical reimbursement amount is 0 and indicates no medical reimbursement has been generated. $Income_{nm}$ indicates that individual n belongs to the first income group m. Here, we divide the family income group into five equal parts. $X_{n}$ denotes control variables. Second, we establish a second-stage result model:

(10) Log(Reimbrusement_amountn)=a0+∑m=15βm×Incomenm+Ynη+ϕn

Here, a general linear model is used to estimate the non-zero medical insurance reimbursement amount, and we assume that the residual terms in Equations (9) and (10) are independent of each other. The other variables in $Y_{n}$ are consistent with the control variables in $X_{n}$.

In the channels that affect the reimbursement of medical insurance, we use the total cost of hospitalization for identification, but there are also additional cases where the total hospitalization cost is 0 in the sample, which contrasts with reality. Here, the case where the total hospitalization cost is 0 defined as the missing value, and when it is greater than 0, it is defined as 1; then, the two models are also used to avoid estimation bias. The model is set as follows:

(11) $$Log(Cost_{n})=a_{0}+\sum_{m=1}^{5}\beta_{m}\times Income_{nm}+Z_{n}\eta +\theta_{n}$$

(12) $$\mathrm{Pr}obit(Cost\_dummy_{n}|Income,X)=a_{0}+\sum_{m=1}^{5}\beta_{m}\times Income_{nm}+X_{n}\eta +\mu_{n}$$

Among them, model $Cost\_dunmmy_{n}$ indicates whether the total medicine cost of hospitalization is greater than 0; $Cost_{n}$ indicates the total medicine cost of hospitalization. The control variables are consistent with Equations (9) and (10).

### 2.3. Data

#### 2.3.1. Data Source

Ethics approval and consent to participate: The authors approve this manuscript and confirmed compliance with human ethics guidelines. Ethics approval committee: National Natural Science Fund of China, August 12, 2019 (approval number: 71904167); and National Social Science Fund of China, June 15, 2018 (approval number: 18AGL018).

The data for this study was from the China Health and Nutrition Survey (CHNS) database, accessed openly to https://www.cpc.unc.edu/projects/china/data. The database covers data from multiple provinces in China, including geographical characteristics, economic development levels, and differences in public resources and health indicators. Ten surveys were conducted between 1989 and 2015, approximately 4400 families visited per survey, and 19,000 individual samples and some community statistics were collected. This paper uses the survey data of 2015. To avoid effects on the statistical results, the missing values and invalid values were eliminated, and we discarded values of the core variables smaller than the 1st percentile or greater than the 99th percentile. The influence of outliers and extreme values on the result estimation may be eliminated. In the end, 1701 valid samples were obtained. The specific variables were selected and processed as follows:

##### Explained Variable

The explained variables of the study are social medical insurance. According to the characteristics of China’s current medical insurance system, including new rural medical insurance, urban residents’ medical insurance, and urban employee medical insurance, we mainly examine the medical insurance for urban and rural residents. For fairness, therefore, we merge rural residents’ and urban residents’ medical insurance to examine the coverage of basic medical insurance. Any item that is insured is recorded as 1 or 0. We express social medical insurance benefits in terms of medical insurance reimbursement ratios. Thus, the insured chooses hospitalization or outpatient services after an illness, and the medical expenses and hospitalization expenses incurred are paid by medical insurance. If it is greater than 0, the insured received the compensation. If it is less than 0, no compensation occurs. The total cost of hospitalization is based on actual hospitalization expenses. For the health status of the insured, we mainly investigate the incidences of hospitalized and chronic diseases. Health care variables refer to preventive health services, such as health checkups, visual acuity tests, blood routine tests, high blood pressure screening, and tumor screening services, which are recorded as 1, or no record as 0. The specific treatment is based on the proportion of actual health care expenditures reimbursed by medical insurance.

##### Explanatory Variables

The study mainly examines the benefit fairness of social medical insurance caused by different household income levels. Therefore, according to the family income variable, it is divided into five samples from low to high.

##### Control Variables

Based on the literature, we mainly control the regional characteristics (Province), family characteristics (Hhsize), and individual characteristics (e.g., Age, Education, Job).

#### 2.3.2. Descriptive Statistics

The selection and definition of the main variables of the article are shown in Table 1. Among them, the core explanatory variables are obtained by dividing income into five equal parts. The average income of each income level is significantly different. Other control variables are shown in Table 1 and will not be explained in detail here.

## 3. Results

### 3.1. Medical Insurance Compensation Level and Hospitalization Possibility

In this study, the Heckman two-stage model was used to estimate the difference between the medical insurance compensation level and the hospitalization possibility of the urban and rural medical insurance groups with different income levels. The corresponding regression results are shown in Table 2; among them, we use the whether the individual is ill as the exclusion variable. The fact that the difference exists between the selection and outcome model and whether the individual is ill affects whether the individual seeks medical treatment but does not affect the specific reimbursement rate. This finding proves that hypothesis 2 of the theoretical derivation is not completely correct.

We use the interaction term between the degree of illness and the income level (Interaction term is degree of illness multiplied by income level) as an instrumental variable, generate a lambda variable in the first, and then bring it into the two-stage Heckman instrumental variable method for testing. The subsequent studies are all based on this processing method. Regarding the control of relevant factors, the results in Table 2 show that the difference in medical compensation is significant. And the IV-Heckman test results show that this conclusion is robust. However, with the highest income group as the reference group, the hospitalization accessibility of other income groups is not significant except the low-income group.

### 3.2. Possible Impact Path

To further explore the fairness of medical compensation for different income groups, we introduce the total cost of hospitalization in the model and use it as the main impact fairness indicator of medical compensation. The third column of Table 3 shows a significant difference among the lowest-income, low-income, high-income with the highest income in terms of total hospitalization costs. And the IV-Heckman test results show that this conclusion is robust. In addition, the middle-income also showed a significant difference with the highest income under the instrumental variable test. The sargan test and first stage F test show that instrument variable selection is appropriate. However, in terms of the applicability of medical insurance, there are no significant differences among different income groups. The results indicate a significant incidence of hospitalization among the lowest-income group, low-income group, middle-income group, high-income group when the various groups participate in social medical insurance. Therefore, hypothesis 1 is established. Jason and Wang et al. observed that the price elasticity of medical expenses (utilization rate) is small and negative, that is, the change in medical compensation increases the utilization rate of individual medical services [41,42]. The main transmission mechanism is the reduction of medical insurance out-of-pocket expenses, and the total hospitalization expenses and medical insurance applicability affect the compensation plan of the system. The results are significant because the effects of transferred payments and private health insurance are considered, but the correctness of this inference is verified in further analysis.

### 3.3. Further Analysis

To examine the net effect of basic medical insurance on different income groups, we combine the restrictions on participation in private medical insurance and social medical assistance in the two-stage model of Heckman to examine the complementary fairness of the completing basic medical insurance system.

#### 3.3.1. Adding Participation in Private Health Insurance in the Model

Participation in private health insurance is a binary restriction variable: participation is 1 and no participation is 0. The estimated results are shown in Table 4. When the conditions for private health insurance are added, the two-stage model of the medical compensation level shows that the medical reimbursement ratios of the middle-income group, the low-income group, and the lowest-income group are all higher than that of the highest income group.

And the IV-Heckman test results show that this conclusion is robust, and also show that other income groups benefit far better than the highest income group. However, the coefficient of each income group in the private health insurance sample is much larger than that in the full sample. This result shows that the overall difference is significant when the impact of private health insurance is not removed, that is, private health insurance as supplementary medical insurance has a greater impact on the other income groups. However, due to the high rate of private health insurance, in theory, the possibility of participating in supplementary medical insurance is low for other income groups, and showing the inefficiency of private health insurance. However, the empirical results contradict it, so hypothesis 2 is not established. In the main impact path, we continue to use the total cost of hospitalization. The results of model (3) in Table 4 show that with the highest income as the reference, the middle-income group, the low-income group, and the lowest income group have significant differences in total cost of hospitalization and have achieved 42%, 63%, and 75%, respectively. The IV-Heckman test results show that this conclusion is robust, but the coefficient is much smaller. One result further indicates that the main transmission path affecting the basic fairness of basic medical insurance is the total cost of hospitalization.

#### 3.3.2. Adding Social Medical Assistance in the Model

In Table 5, model (1) and model (2) are estimates of the level of medical compensation. The results show that after the introduction of medical social assistance, with the highest income group as reference, the lowest income group significantly differ, with the highest income group in the reimbursement proportion of medical insurance. And the IV-Heckman test results show that this conclusion is robust, and it also shows that there is a significant difference in reimbursement for low-income groups. The medical reimbursement ratio of the lowest-income group is 45%, and 29% higher than the highest income group, and according to the IV-Heckman model, the ratio is 525%, and 458% higher.

This result shows that social medical assistance affects income groups below the middle income. It provides financial support for its direct medical expenses. Additionally, it improves its health level through other paths, such as direct health education, publicity, and third-party medical institution assistance, and has the greatest impact on the lowest income group. The results of model (4) in Table 5 show that after the introduction of social medical assistance restrictions, with the highest income group as the reference group, the high-income group, middle-income group, low-income group, and lowest income group have significant differences in total hospitalization costs: 32%, 30%, 30%, and 29% higher than the highest income group, respectively. These results indicate that the total medical expenses are the main path affecting the supplementary fairness of medical insurance, but that the high income group has the highest total medical expenses.

## 4. Discussion

The regression results after further adding the constraints to the model show that the differences between different income groups are significant, and this result further indicates that among the different income groups, because of the impact of supplementary medical insurance (private health insurance) and social assistance, the overall medical fairness of the high-income group, low-income group, and the lowest income group greatly improves. We also observe that compared with the highest-income group, below the middle-income groups have obvious insufficient guarantees in the fairness of medical compensation and rely more on their medical insurance, showing greater group differences.

The results of this estimate in the study are verified. Notably, the analysis has limitations, especially regarding the development of the social economy; namely, people’s medical expenditure is no longer purely medical expenses but includes preventive health expenditures. Scholars have also conducted studies that are in-depth regarding the relationship between preventive health care and medical expenditure, including health differentiation and health inequalities in the context of the differences in medical expenditure levels and health prevention mechanisms operation and health recovery mechanisms that affect health inequalities. The construction of a universal health care system coordinated by the needs of health care and long-term care expenditure was observed, and the construction of a health care system from a social welfare perspective was also observed [34,35,43]. Therefore, we further introduce health care expenditure as a core explanatory variable to expand the estimation results in the literature. In this study, health care refers to the actual level of health care expenditure based on health prevention expenditures.

We further study the fairness of compensation for different income groups on the basis of health care (Table 6 and Table 7). Both Table 6 and Table 7 are based on IV-Heckman test results. The statistical results in Table 6 show significant differences in the level of health care compensation among the different populations, and the maximum coefficient difference is observed in health care compensation between the lowest income group and the highest one. This result indicates a significant difference in income and compensation for health care.

The last four columns of Table 6 are IV-Heckman two-stage regression models with constraints added. The result shows that the supplementary private health insurance enhances the level of medical insurance compensation among the other groups. And the maximum coefficient difference is also observed between the lowest income group and the highest one. The coefficients remain positive, indicating that private health insurance also has a positive impact on health care compensation for the other groups. This result is reflected in model (6), introducing the limitation conditions of social medical assistance. For example, in model (6) of Table 6, the compensation level of medical care for the low income group and middle income group are significantly higher than the highest income group (11.26, 33.72), which further validates the aforementioned analysis, that is, compensatory medical social assistance promotes the level of health care compensation for the below middle-income group.

In terms of the main transmission paths of group differences in medical reimbursement, we also introduce the total cost of medical care to test the results (Table 7). The results of model (4) in Table 7 show that the significant differences in the total medical and health care costs of the other groups are higher than the highest income group, respectively, when using whether to participate in private health insurance as the two-stage restriction, with the highest income group as the reference group. The results of model (6) show that the other groups do not significantly differ from the highest income group, when social assistance is used as the two-stage restriction and the highest income group as the reference group. The aforementioned results show that in the aspect of medical insurance compensation, medical expenditure is also the main transmission path to inter-group equity. Additionally, it further shows the compensation differences of basic medical expenditure in five income groups. Differences deepen the inequity of hospitalization expenditure.

## 5. Conclusions

The basic medical insurance system under the concept of equality provides a basic theoretical framework in this study. That is, when the system is the same, the insured of different incomes receive the same premium, assuming that their needs for health service are the same. However, the difference in compensation for medical insurance is problematic regarding group benefit fairness because of in-patient health service accessibility or applicability of medical insurance.

The two-stage Heckman model test results show that (1) when the insured group does not have social medical assistance and the insured group participates in private health insurance is excluded, an obvious difference is observed among different income groups, but the coefficient difference is small. (2) In terms of the income coefficient of medical expenditure, the higher the income, the more the medical consumption of the high-income group, and the lower the income elasticity of the medical consumption of the high-income group. Therefore, further analysis shows that (3) under the restriction of private health insurance, significant differences are observed among the groups except the highest income group. Under the restriction of social medical assistance, 80% of the income groups below the highest income have significant differences, and the level of medical compensation and total hospitalization expenses are significantly higher than the highest income group. To test the reliability of the results and the underlying reasons for the difference in fairness of medical insurance compensation level, the further test results show that (4) the fairness of basic medical compensation is similar, and the compensation level of medical insurance presents the same significance. After introducing restrictions into the two-stage model, the results remain robust, showing that the difference in the compensation level of preventive health care is the indirect cause compared with the basic medical care compensation. The amount of medical expenditure directly affects the difference in the health level of the group, which reflects the difference between total medical expenses and the medical compensation level. However, for the lowest income group, due to the income threshold effect of supplementary medical insurance and inequities in preventive health care, it shows a significant difference in the actual level of medical compensation. In the sample of medical social assistance, the difference in preventive health care of different income groups does not significantly prove the accuracy of this conclusion.

In the process of optimizing the basic medical insurance system, policies not only further strengthen the fairness adjustment to the medical insurance compensation level for different income groups, but also improve the basic medical equity of different income groups and appropriate supplementary system intervention. In the basic medical insurance systems, the middle-income group often does not benefit from the policy, but in the pure market environment, such groups are not. Therefore, policies for supplementary systems should avoid institutional gaps and strengthen medical security for middle-income groups. For example, the reimbursement ratio of medical expenditure should be increased, and the catalog of medical care expenditure should be enlarged to attain the supplementary fairness of basic medical insurance.

## Figures and Tables

**Table 1:** Variable descriptive statistics.

Variable	Sample	Definition	Mean	S.D.	Min	Max
Lowest income: 20%	1701	The per capita income of the family is between 0 and 5000 yuan, and the average income in this period is 2142.26 yuan	0.20	0.40	0	1
Low income: 20%	1701	The per capita income of the family is between 5000 and 12,000 yuan, and the average income in this period is 8758.66 yuan	0.20	0.41	0	1
Middle income: 20%	1701	The per capita income of the family is between 12,000 and 20,000 yuan, and the average income in this period is 15,880.97 yuan	0.20	0.39	0	1
High income: 20%	1701	The family’s per capita income is between 20,000 and 32,800 yuan, and the average income in this period is 25,857.47 yuan	0.20	0.40	0	1
Highest income: 20%	1701	The per capita income of the family is between 32,800 and 56,476 yuan, and the average income of this segment is 45,891.88 yuan	0.20	0.40	0	1
Medicare Reimbursement Ratio	1701	Proportion of actual medical compensation received	32.51	37.07	0	100
lnincome	1701	1% shrinking of income, and take the logarithm	5.75	2.01	1.10	11.51
social medical insurance	1701	Participation in social medical insurance = 1, not participation = 0	0.74	0.44	0	1
private medical insurance	1701	Participating in private medical insurance = 1, not participating = 0	0.37	0.48	0	1
social medical assistance	1701	Received social medical assistance = 1, not received = 0	0.08	0.26	0	1
hhsize	1701	Actual population size in household survey year	3.85	1.72	1	15
job	1701	Participating in work = 1, not participating = 0	0.47	0.50	0	1
highedu	1701	Elementary school = 1, junior high school = 2, high school = 3, secondary vocational learning = 4, college or university = 5, master’s degree and above = 6	2.62	1.35	1	6
age	1701	Actual individual age in survey year	44.44	21.58	8	100
age2	1701	Age squared	2440.7	1824	64	10,000
huzhu	1701	Householder = 1, No = 0	0.14	0.35	0	1
married	1701	Married = 1, unmarried = 0	0.30	0.46	0	1
whether or not ill	1701	ill = 1, not ill = 0	0.11	0.32	0	1
degree of illness	1701	not serious = 1, general = 2, quite serious = 3	1.68	0.66	1	3

**Table 2:** Medical insurance compensation level and hospital accessibility.

	Medicare Reimbursement Ratio				Hospitalization Possibility (3) IV-Probit
	(1) Heckman		(2) IV-Heckman		
	First Part (Probit)	Second Part (GLM)	First Part (Probit)	Second Part (2SLS)	
Lowest income 20%	−28.30 ***	−0.14 **	−6.74	6.62 ***	−0.45
	(3.55)	(0.06)	(6.78)	(0.20)	(0.34)
Low income 20%	−24.57 ***	−0.15 **	−5.26	5.78 ***	−0.61 *
	(3.50)	(0.06)	(5.83)	(0.18)	(0.34)
Middle income 20%	−20.08 ***	−0.14 **	−3.54	5.43 ***	−0.51
	(3.38)	(0.06)	(5.43)	(0.17)	(0.35)
High income 20%	−12.11 ***	−0.26 ***	−2.02	4.33 ***	−0.53
	(3.27)	(0.06)	(4.37)	(0.13)	(0.35)
Lambda	-	-	−35.51 *** (11.47)	11.54 *** (0.34)	-
/mills ratio	−5.56 *** (1.72)		-	-	-
Sample	917	300	917	300	1128
DWH/Wald test	3.98 *** (*p* = 0.00)		3.98 *** (*p* = 0.00)		0.10 (*p* = 0.75)
Sargan test	-		8.46 (*p* = 0.36)		-
First stage F	-		511.37 (*p* = 0.00)		-

Note: *, **, *** indicate significant levels at 10%, 5%, and 1%, respectively. Control variables are not listed. The variance expansion factor measurement results show that the mean value of the variance factor VIF of the independent variable is 1.42, the largest is 1.9 of the lowest income group, which is far less than the critical value of 10, indicating that there is no multiple colinearity. The instrument variables are processed by the model. The wald test results show that the instrument variables are valid; the control variables are not listed. The following table is the same as the definition here.

**Table 3:** Total hospitalization expenses and the applicability of medical insurance.

	Total Hospitalization Costs				Applicability of Medical Insurance (3) IV-Probit
	(1) Heckman		(2) IV-Heckman		
	First Part (OLS)	Second Part (GLM)	First Part (OLS)	Second Part (2SLS)	
Lowest income 20%	−0.17	−0.16 **	−0.26	0.30 ***	0.22
	(0.25)	(0.08)	(0.30)	(0.01)	(0.37)
Low income 20%	0.39	−0.16 **	0.42	0.29 ***	−0.56
	(0.24)	(0.08)	(0.30)	(0.01)	(0.89)
Middle income 20%	0.16	−0.09	0.16	0.29 ***	0.75
	(0.24)	(0.08)	(0.28)	(0.01)	(0.21)
High income 20%	0.23	−0.26 ***	0.09	0.32 ***	0.67
	(0.27)	(0.08)	(0.32)	(0.02)	(0.19)
lambda	-	-	−9202.1 ** (3965.9)	3903.9 *** (1421.9)	-
/mills ratio	−0.56 *** (0.11)		-	-	-
Sample	591	300	591	300	897
DWH/Wald test	-		0.60 *** (0.00)		0.04 (*p* = 0.85)
Sargan test	-		62.55 (0.45)		-
First stage F	-		744.71 (0.00)		-

Note: ** *p* < 0.05, *** *p* < 0.01.

**Table 4:** Medical insurance compensation level and total hospitalization expenses with restrictions on private health insurance.

	Medicare Reimbursement Ratio				Total Hospitalization Costs			
	(1) Heckman		(2) IV-Heckman		(3) Heckman		(4) IV-Heckman	
	First Part (Probit)	Second Part (GLM)	First Part (probit)	Second Part (2SLS)	First Part (OLS)	Second Part (GLM)	First Part (OLS)	Second Part (2SLS)
Lowest income 20%	−29.36 ***	0.75 ***	−7.57	5.81 ***	−0.54 *	0.75 ***	−0.26	0.30 ***
	(4.55)	(0.20)	(6.35)	(0.24)	(0.28)	(0.21)	(0.28)	(0.01)
Low income 20%	−25.99 ***	0.63 ***	−6.066	6.594 ***	0.04	0.63 ***	0.394	0.299 ***
	(4.41)	(0.18)	(5.63)	(0.28)	(0.27)	(0.19)	(0.29)	(0.01)
Middle income 20%	−24.39 ***	0.38 **	−5.41	5.82 ***	−0.08	0.42 **	0.15	0.30 ***
	(4.37)	(0.18)	(5.82)	(0.24)	(0.27)	(0.18)	(0.29)	(0.01)
High income 20%	−13.60 ***	−0.04	−0.824	5.51 ***	0.14	−0.04	0.06	0.33 ***
	(4.58)	(0.17)	(5.74)	(0.23)	(0.29)	(0.17)	(0.32)	(0.02)
Lambda2/Lambda3	-	-	−32.39 ***	9.79 ***	-	-	−10,046.3 **	4223.2 ***
	-	-	(10.07)	(0.37)	-	-	(4917.6)	(1282.1)
/mills ratio	−10.88 (7.58)		-	-	−1.63 *** (0.44)		-	-
DWH	-		3.19 ** (*p* = 0.01)		-		0.37 *** (*p* = 0.00)	
Sargan test	-		0.24 (*p* = 0.62)		-		63.01 (*p* = 0.28)	
First stage F	-		487.61		-		749.60	
Sample	580	300	580	300	572	300	572	300

Note: * *p* < 0.1, ** *p* < 0.05, *** *p* < 0.01.

**Table 5:** Medical insurance compensation level and total hospitalization expenses with social medical assistance limits.

	Medicare Reimbursement Ratio				Total Hospitalization Costs			
	(1) Heckman		(2) IV-Heckman		(3) Heckman		(4) IV-Heckman	
	First Part (Probit)	Second Part (GLM)	First Part (Probit)	Second Part (2SLS)	First Part (OLS)	Second Part (GLM)	First Part (OLS)	Second Part (2SLS)
Lowest income 20%	−18.02	0.54 **	4.12	5.25 ***	−0.11	0.55 **	−0.34	0.29 ***
	(22.50)	(0.27)	(5.09)	(0.27)	(1.29)	(0.27)	(0.29)	(0.01)
Low income 20%	−8.94	0.29	5.51	4.58 ***	1.01	0.30	0.42	0.30 ***
	(21.36)	(0.28)	(5.0)	(0.22)	(1.20)	(0.28)	(0.30)	(0.01)
Middle income 20%	23.85	0.05	2.31	4.17	0.86	0.06	0.14	0.30 ***
	(24.47)	(0.31)	(4.62)	(0.59)	(1.37)	(0.31)	(0.29)	(0.01)
High income 20%	−25.42	0.26	6.42	1.09	0.22	0.26	0.07	0.32 ***
	(21.34)	(0.28)	(14.27)	(0.65)	(1.19)	(0.28)	(0.324)	(0.02)
Lambda4/Lambda5	-	-	−15.59 ***	4.87 ***	-	-	15.10	18.66 ***
	-	-	(4.94)	(0.23)	-	-	(27.32)	(5.38)
/mills ratio	3.80 (13.53)		-	-	0.03 (0.72)		-	-
Sample	788	338	587	300	591	300	591	300
DWH	-		0.09 *** (*p* = 0.00)		-		0.31 ** (*p* = 0.03)	
Sargan test	-		0.37 (*p* = 0.83)		-		74.62 (*p* = 0.57)	
First stage F	-		65.72		-		760.72	

Note: ** *p* < 0.05, *** *p* < 0.01.

**Table 6:** Medical care compensation levels with different incomes and estimation of introducing restrictive variables.

	IV-Heckman Two-Stage Model					
	Insured Sample		Private Health Insurance		Social Medical Assistance	
	(1) First Part (Probit)	(2) Second Part (2SLS)	(3) First Part (Probit)	(4) Second Part (2SLS)	(5) First Part (Probit)	(6) Second Part (2SLS)
Lowest income 20%	−7.222	17.74 ***	−10.45	26.86 ***	−30.11	21.74
	(19.77)	(2.68)	(29.08)	(5.23)	(30.06)	(14.31)
Low income 20%	−3.608	11.22 ***	−5.60	16.25 ***	5.39	11.26 ***
	(12.38)	(1.33)	(17.84)	(2.88)	(12.40)	(2.293)
Middle income 20%	−2.590	10.34 ***	−0.681	10.34 ***	52.00	33.72 *
	(10.47)	(1.15)	(10.74)	(1.20)	(45.28)	(17.79)
High income 20%	5.981	8.20 ***	5.58	9.34 ***	48.07	29.35
	(9.65)	(0.69)	(8.73)	(0.98)	(44.15)	(22.10)
lambda6/lambda7/lambda8	−1.090	25.90 ***	5.50	45.16 ***	111.2	76.03
	(27.93)	(3.62)	(46.78)	(8.96)	(112.0)	(52.88)
Sample	788	155	587	144	591	89
DWH	4.82 *** (*p* = 0.00)		2.33 * (*p* = 0.07)		7.21 *** (*p* = 0.00)	
Sargan test	0.89 (*p* = 1.67)		3.56 (*p* = 0.54)		2.18 (*p* = 2.74)	
First stage F	22.70		49.13		154.39	

Note: * *p* < 0.1, *** *p* < 0.01.

**Table 7:** Estimation of health care costs with different income and introduction of restricted variables.

	IV-Heckman Two-Stage Model					
	Insured Sample		Private Health Insurance		Social Medical Assistance	
	(1) First Part (OLS)	(2) Second Part (2SLS)	(3) First Part (OLS)	(4) Second Part (2SLS)	(5) First Part (OLS)	(6) Second Part (2SLS)
Lowest income 20%	−82.47	91.14 ***	−74.97	122.6 ***	−100.2	132.1
	(79.79)	(18.36)	(102.4)	(31.06)	(974.8)	(37069.1)
Low income 20%	−42.54	56.78 ***	−33.30	72.80 ***	−84.65	65.72
	(51.12)	(10.57)	(60.62)	(16.03)	(203.7)	(7286.3)
Middle income 20%	52.14	52.96 ***	13.66	46.92 ***	86.90	206.5
	(58.93)	(9.37)	(40.19)	(9.45)	(2116.8)	(80,925.3)
High income 20%	77.17	41.33 ***	80.52	42.66 ***	−22.30	176.8
	(56.71)	(6.35)	(61.63)	(7.85)	(1402.7)	(53,698.2)
lambda6/lambda7/lambda8	94.14	132.8 ***	91.41	204.9 ***	55.15	463.4
	(122.7)	(25.66)	(174.5)	(51.78)	(3870.7)	(148,055.4)
Sample	657	161	573	148	386	94
DWH	9.31 * (*p* = 0.06)		0.83 *** (*p* = 0.00)		20.51 *** (*p* = 0.00)	
Sargan test	2.69 (*p* = 1.32)		0.64 (*p* = 0.17)		8.56 (*p* = 0.58)	
First stage F	14.37		32.79		253.61	

Note: * *p* < 0.1, *** *p* < 0.01.
